# Independent prognostic role of human papillomavirus genotype in cervical cancer

**DOI:** 10.1186/s12879-017-2465-y

**Published:** 2017-06-05

**Authors:** Dong Hang, Meiqun Jia, Hongxia Ma, Jing Zhou, Xiaoshuang Feng, Zhangyan Lyu, Jian Yin, Hong Cui, Yin Yin, Guangfu Jin, Zhibin Hu, Hongbing Shen, Kai Zhang, Ni Li, Min Dai

**Affiliations:** 1 Department of Epidemiology and Biostatistics, School of Public Health, Nanjing Medical University, Nanjing, 211166 China; 2 Program Office for Cancer Screening in Urban China, National Cancer Centre/Cancer Hospital, Chinese Academy of Medical Sciences and Peking Union Medical College, Beijing, 100021 China; 3 State Key Laboratory of Reproductive Medicine, Nanjing Medical University, Nanjing, 211166 China; 4 Department of Cancer Prevention, National Cancer Centre/Cancer Hospital, Chinese Academy of Medical Sciences and Peking Union Medical College, Beijing, 100021 China

**Keywords:** Cervical cancer, Prognosis, Human papillomavirus, Genotype

## Abstract

**Background:**

Although the correlation of HPV genotype with cervical precursor lesions and invasive cancer has been confirmed, the role of HPV genotype in cervical cancer prognosis is less conclusive. This study aims to systematically investigate the independent prognostic role of HPV genotype in cervical cancer.

**Methods:**

A total of 306 eligible patients provided cervical cell specimens for HPV genotyping before therapy and had a median follow-up time of 54 months after diagnosis. Survival times were measured from the date of diagnosis to the date of cervical cancer-related death (overall survival, OS) and from the date of diagnosis to the date of recurrence or metastasis (disease free survival, DFS). Log-rank tests and Cox proportional hazard models were performed to evaluate the association between HPV genotype and survival times.

**Results:**

A total of 12 types of high-risk HPV were detected and the leading ten types belong to two species: alpha-9 and alpha-7. HPV16 and 18 were the two most common types, with the prevalence of 60.8% and 8.8%, respectively. In the univariate analysis, HPV16-positive cases were associated with better OS (*P* = 0.037) and HPV16-related species alpha-9 predicted better OS and DFS (both *P* < 0.01). After adjusting for age, FIGO stage, and therapy, HPV16 showed a hazard ratio (HR) of 0.36 (95% CI: 0.18, 0.74; *P* = 0.005) for OS, and alpha-9 resulted in a HR of 0.17 (95% CI: 0.08, 0.37; *P* < 0.001) for OS and 0.32 (95% CI: 0.17, 0.59; *P* < 0.001) for DFS.

**Conclusions:**

HPV genotype poses differential prognoses for cervical cancer patients. The presence of HPV16 and its related species alpha-9 indicates an improved survival.

**Electronic supplementary material:**

The online version of this article (doi:10.1186/s12879-017-2465-y) contains supplementary material, which is available to authorized users.

## Background

Cervical cancer is the fourth most common malignancy in females worldwide, with an estimated 527,600 new cases and 265,700 deaths per year [1]. The etiological relationship has been well established between human papillomavirus (HPV) and cervical cancer. Recently, more than 170 HPV genotypes have been identified and classified according to their L1 open reading frame [2]. When HPVs have 60–70% genomic nucleotide similarity, they are clustered into the same species. Two HPV species, alpha-7 (HPV18, 39, 45, 59, 68, and 70) and alpha-9 (HPV16, 31, 33, 35, 52, 58, and 67), are responsible for over 80% of all cervical cancer cases [3].

Although there has been much evidence on the role of HPV genotype in cervical precursor lesions and invasive cancer, it remains unclear whether they affect prognosis of cervical cancer. Furthermore, existing results on the relationship of HPV genotype with survival are heterogeneous. For example, early evidence showed that HPV16 positivity predicted poor prognosis and was associated with histological features of prognostic significance such as squamous cell carcinomas, pelvic node metastases, and lymphatic space invasion [4]. But some studies reported that HPV18 positivity, rather than HPV16, is a poor prognostic factor [5, 6]. Histologic type of adenocarcinomas, pelvic lymph node metastasis, and deeper stromal invasion was more common in HPV18-caused cervical cancer [6]. In addition, HPV31-related and HPV58-related types were found to be associated with better survival outcome [7, 8]. However, no prognostic value of HPV type was reported by the other studies [9, 10]. The inconsistency may be attributed to the significant differences in sample size, length of follow-up, assay methods, and adjustment for known prognostic factors.

To better understand the role of HPV genotype in prognosis of patients with cervical cancer, we assessed the association of HPV genotype with overall survival (OS, the time between the date of diagnosis and the date of cervical cancer-related death) and disease free survival (DFS, the time between the date of diagnosis and the date of recurrence, distant metastasis, or the last follow-up) among 306 cases of cervical cancer from China.

## Methods

### Patients

Cervical cancer patients were consecutively recruited from Cancer Hospital, Chinese Academy of Medical Sciences from 2010 to 2012. We included patients who had a first diagnosis of histologically confirmed invasive cervical cancer, and the sampling of cervical exfoliated cells for HPV genotyping were taken by a gynecologist before therapy. Patients were excluded for the following criteria: a history of hysterectomy or conization, recurrent cervical cancer, other preexisting malignancies, and those with less than two months of survival after completing therapy. Patient’s age, FIGO stage, tumor histology, and pathologic variables were retrieved from medical records. This study was approved by the ethics committees of National Cancer Centre/Cancer Hospital, Chinese Academy of Medical Sciences, and all patients provided informed written consent before study enrollment.

### HPV genotyping

Genomic DNA was extracted from cervical cell specimens manually by using QIAamp DNA Mini Kit, according to the manufacture’s protocol (Qiagen, Valencia, CA, USA). The quality of extracted DNA was assessed by PCR with a set of primers for the housekeeping gene β-actin (forward primer, 5′-GAAATCGTGCGTGACATTAA-3′; reverse primer 5′-AAGGAAGGCTGGAAGA.

GTG-3′). All β-actin positive specimens were tested for HPV DNA by following the manufacturer’s protocol of a HPV GenoArray Test Kit (HybriBio, Beijing, China), which is a Chinese FDA-approved assay for HPV genotyping. A total of 21 HPV types could be detected simultaneously, including 13 high-risk (HR) types (HPV16, 18, 31, 33, 35, 39, 45, 51, 52, 56, 58, 59, and 68), two intermediate-risk types (HPV53 and 66), and six low-risk HPV types (HPV6, 11, 42, 43, 44, and 81).

### Treatment and follow up

Treatment information was retrieved from medical records and was summarized and grouped as follows: surgery alone (radical hysterectomy and pelvic lymphadenectomy); surgery plus adjunctive chemotherapy (CT), radiotherapy (RT) or chemoradiotherapy (CRT); concurrent chemoradiotherapy (CCRT); CT or RT only. Each patient was followed up every 3 months in the first year and every 6 months in the next years by personal or family contacts, until June 2016. Hospital medical records were obtained in order to confirm the reported events. Only validated events were included in analysis. Overall survival (OS) was defined as the time between the date of diagnosis and the date of cervical cancer-related death or the last follow-up. Disease-free survival (DFS) was measured from the date of diagnosis to the date of recurrence, distant metastasis, or the last follow-up.

### Statistical analysis

The data were analyzed by using the Stata version 11.0 (Stata Corporation, Texas, USA). To assess the potential of HPV type as a prognostic biomarker for cervical cancer patients with no matter single or multiple infections, all cases were included for HPV16 and 18 survival analysis. Multiple infections with only alpha-9 types were included for alpha-9 survival analysis. Survival curves were generated using the Kaplan-Meier method, and comparisons were performed using the log–rank test. Multivariate analyses of the factors associated with OS and DFS were done using Cox proportional hazard regression model. In the stratified analysis, the chi-square test-based Q-statistic was applied to test the heterogeneity between subgroups defined by age, FIGO stage, and treatment. All *P* values presented were two-sided and were assumed significant as *P* < 0.05.

## Results

### Patient characteristics

The demographic and clinical characteristics of cervical cancer patients are summarized in Table 1. This study included 306 women with a median age of 48 years (range: 26–71 years). The most common histological type was squamous cell carcinoma (96.7%) and the others were adenocarcinoma (AC) and adeno-squamous carcinoma (ASC). Most of patients were diagnosed with FIGO stage I-II (81.0%). Seventy-eight patients (25.5%) received surgery, and 21 (6.9%) surgery plus CT/RT/CRT, 160 (52.3%) CCRT, 47 (15.4%) CT or RT only.

**Table 1:** Characteristics of cervical cancer patients

Characteristics	Patients (*N* = 306)	
	No.	%
Age, years		
Median	48	
Range	26–71	
< 40	45	14.71
40–50	133	43.46
> 50	128	41.83
Histologic type^a^		
SCC	296	96.73
AC/ASC	10	3.26
FIGO stage		
I	105	34.31
II	143	46.73
III	52	16.99
IV	6	1.96
Differentiation		
Poor	87	28.43
Moderate	126	41.18
Well	11	3.59
Unclassified	82	26.80
Treatment^b^		
Surgery	78	25.49
Surgery plus CT/RT/CRT	21	6.86
CCRT	160	52.29
CT or RT only	47	15.36

^a^ *SCC* squamous cell carcinoma, *AC* adenocarcinoma, *ASC* adeno-squamous carcinoma

^b^ *CT* chemotherapy, *RT* radiotherapy, *CRT* chemoradiotherapy, *CCRT* concurrent chemoradiotherapy

### HPV genotypes

A total of 12 types of high-risk HPV were detected in this study (Table 2). The top three types were HPV16 (60.8%), 18 (8.8%), and 52 (5.9%). In the leading ten types, five (HPV16, 52, 33, 31, 58) were alpha-9 and the others (HPV18, 39, 59, 68, 45) were alpha-7. Of 306 patients, 268 (87.6%) harbored single-type and 38 (12.4%) contained multiple-type infections. As shown in Additional file 1: Table S1, HPV16 (81.6%, 31/38) and 52 (42.1%, 16/38) were the predominant types that made up the multiple infections.

**Table 2:** Distribution of HR-HPV types in cervical cancer patients

HR-HPV type^a^		Species	SCC^b^ (*N* = 296)	AC/ASC^c^ (*N* = 10)	Total (%)
Single infection					268 (87.58)
	16	alpha-9	184	2	186 (60.78)
	18	alpha-7	24	3	27 (8.82)
	52	alpha-9	17	1	18 (5.88)
	39	alpha-7	8	0	8 (2.61)
	33	alpha-9	6	0	6 (1.96)
	31	alpha-9	5	0	5 (1.63)
	58	alpha-9	4	1	5 (1.63)
	59	alpha-7	4	0	4 (1.31)
	68	alpha-7	4	0	4 (1.31)
	45	alpha-7	3	0	3 (0.98)
	51	alpha-5	1	0	1 (0.33)
	56	alpha-10	1	0	1 (0.33)
Multiple infection			35	3	38 (12.42)
Alpha-7 only			43	3	46 (15.03)
Alpha-9 only			237	4	241 (78.76)

^a^ *HR-HPV* high risk human papillomavirus

^b^ *SCC* squamous cell carcinoma.

^c^ *AC* adenocarcinoma, *ASC* adeno-squamous carcinoma

### Survival analysis

The mean number of follow-up was 5 for each patient and the median time for these follow ups was 54 (range, 3–75) months. A total of 58 patients (19.0%) had experienced treatment failure, including 27 recurrences and 38 distant metastases (7 patients had both). In addition, 34 deaths (11.1%) were attributed to cervical cancer. The 5-year OS rate for the entire cohort was 87.1% (95% CI: 82.1–90.8%), and the corresponding DFS rate was 78.3% (95% CI: 72.5–83.1%).

**Fig. 1:**
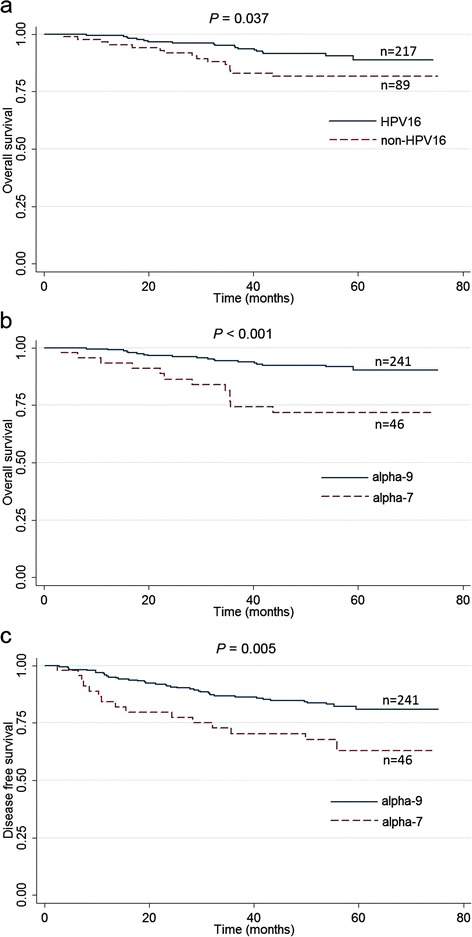
The role of HPV genotype in cervical cancer prognosis. **a** Kaplan-Meier overall survival (OS) curves for HPV16 and non-HPV16 types; (**b**) Kaplan-Meier OS curves for the alpha-7 and alpha-9 species; (**c**) Kaplan-Meier disease free survival (DFS) curves for the alpha-7 and alpha-9 species

In univariate analysis (Table 3), FIGO stage IV was significantly associated with poorer OS (*P* < 0.001) and DFS (*P* < 0.001), while primary surgical treatment was associated with a better OS (*P* = 0.004) and DFS (*P* = 0.019). Of note, patients infected with HPV16 had a better OS than those with any other types (*P* = 0.037) (Fig. 1a). HPV16-related species alpha-9 also posed a better OS (*P* < 0.001) and DFS (*P* = 0.005), compared to alpha-7 (Fig. 1b and c). No significant association with prognosis was found for HR-HPV multiple infections, HPV18 and the other types.

**Table 3:** Univariate and multivariate analyses of prognostic factors for cervical cancer patients

Variable		Overall survival					Disease free survival				
		3-Year Rate	5-Year Rate	*P* ^e^	Adjusted HR	*P* ^b^	3-Year Rate	5-Year Rate	*P* ^e^	Adjusted HR	*P* ^f^
		(95% CI)	(95% CI)		(95% CI) ^f^		(95% CI)	(95% CI)		(95% CI)^f^	
Age, years											
	< 40	93.18 (80.33, 97.75)	93.18 (80.33, 97.75)	0.634	1.00		86.43 (72.24, 93.67)	78.58 (62.60, 88.34)	0.956	1.00	
	40–50	91.20 (84.67, 95.03)	85.39 (76.26, 91.21)		1.74 (0.49, 6.20)	0.390	83.84 (76.07, 89.26)	78.48 (68.81, 85.46)		0.72 (0.32, 1.60)	0.417
	> 50	91.75 (85.20, 95.47)	86.99 (79.22, 92.00)		1.09 (0.30, 4.04)	0.893	87.78 (80.55, 92.45)	78.01 (68.46, 84.99)		0.56 (0.25, 1.30)	0.177
Histologic type^a^											
	SCC	91.77 (87.87, 94.46)	87.07 (81.97, 90.81)	0.943			85.79 (81.13, 89.37)	77.96 (71.96, 82.82)	0.478		
	AC/ASC	87.50 (38.70, 98.14)	87.50 (38.70, 98.14)				87.50 (38.70, 98.14)	87.50 (38.70, 98.14)			
FIGO stage^b^											
	I	95.82 (89.23, 98.41)	94.73 (87.79, 97.77)	**<0.001**	1.00		93.83 (86.78, 97.18)	86.60 (75.90, 92.76)	**<0.001**	1.00	
	II	95.68 (90.63, 98.04)	88.83 (79.72, 94.00)		0.98 (0.27, 3.50)	0.970	89.86 (83.48, 93.87)	84.14 (76.10, 89.65)		1.20 (0.43, 3.36)	0.735
	III	74.66 (59.67, 84.76)	70.20 (54.87, 81.17)		3.40 (0.92, 12.52)	0.066	66.66 (51.44, 78.08)	53.52 (37.74, 66.96)		4.54 (1.53, 13.42)	**0.006**
	IV	60.00 (12.57, 88.18)	40.00 (5.20, 75.28)		9.91 (1.87, 52.39)	0.007	16.67 (0.77, 51.68)	16.67 (0.77, 51.68)		13.21 (3.24, 53.82)	**<0.001**
Differentiation											
	Poor	90.36 (81.64, 95.07)	86.83 (76.49,92.82)	0.383			83.23 (73.31, 89.71)	68.83 (55.26, 79.03)	0.068		
	Moderate	90.68 (83.80, 94.73)	88.08 (80.70,92.77)				88.27 (80.98, 92.88)	82.83 (73.70, 89.02)			
	Well	90.91 (50.81, 98.67)	72.73 (37.08,90.28)				63.64 (29.69, 84.52)	63.64 (29.69, 84.52)			
	Unclassified										
Treatment^§^											
	Surgery	100	98.53 (90.02, 99.79)	**0.004**	1.00		96.05 (88.24, 98.71)	84.25 (66.36, 93.09)	**0.019**	1.00	
	Surgery plus CT/RT/CRT	100	93.75 (63.23, 99.10)		3.98 (0.25, 64.19)	0.331	95.00 (69.47, 99.28)	95.00 (69.47, 99.28)		0.43 (0.52, 3.56)	0.433
	CCRT	90.02 (83.99, 93.87)	82.55 (74.02, 88.49)		8.26 (0.87, 78.75	0.066	83.34 (76.34, 88.43)	73.83 (65.27, 80.60)		1.44 (0.44, 4.74)	0.545
	CR or RT	79.55 (64.38, 88.79)	79.55 (64.38, 88.79)		13.91 (1.59, 121.54)	0.017	73.37 (57.89, 83.91)	73.37 (57.89, 83.91)		1.92 (0.63, 5.88)	0.253
HPV16											
	Negative	83.09 (73.10, 89.63)	81.77 (71.57, 88.60)	**0.037**	1.00		82.89 (72.81, 89.50)	75.71 (64.69, 83.71)	0.342		
	Positive	95.17 (91.21, 97.37)	88.93 (82.31, 93.17)		**0.36 (0.18, 0.74)**	**0.005**	87.07 (81.70, 90.95)	79.03 (71.40, 84.84)			
HPV18											
	Negative	91.87 (87.80, 94.62)	87.58 (82.23, 91.40)	0.404			86.49 (81.69, 90.11)	78.76 (72.58, 83.71)	0.69		
	Positive	90.04 (72.14, 96.69)	83.12 (64.02, 92.62)				80.85 (62.18, 90.93)	74.63 (52.38, 87.60)			
HR-HPV species^d^											
	Alpha-7	74.38 (58.47, 84.93)	71.81 (55.65, 82.94)	**<0.001**	1.00		76.32 (60.41, 86.51)	62.96 (45.04, 76.47)	**0.005**	1.00	
	Alpha-9	94.34 (90.44, 96.67)	90.42 (84.82, 94.02)		**0.17 (0.08, 0.37)**	**<0.001**	87.89 (82.93, 91.47)	80.88 (74.33, 85.92)		**0.32 (0.17, 0.59)**	**<0.001**
HR-HPV pattern											
	Single	90.90 (86.61, 93.86)	87.04 (81.66, 90.94)	0.939			85.47 (80.51, 89.25)	77.41 (71.06, 82.53)	0.389		
	Multiple	97.30 (82.32, 99.61)	87.77 (70.52, 95.24)				88.83 (72.85, 95.67)	85.54 (68.53, 93.75)			

^a^SCC, squamous cell carcinoma; AC, adenocarcinoma; ASC, adeno-squamous carcinoma

^b^FIGO, International Federation of Gynecology and Obstetrics

^c^CT, chemotherapy; RT, radiotherapy; CRT, chemoradiotherapy; CCRT, concurrent chemoradiotherapy

^d^HR-HPV, high risk human papillomavirus

^e^Calculated by using the log-rank test. Bold values indicate *P* < 0.05

^f^Adjusted for age, FIGO stage, and treatment in the Cox regression models

In multivariate models with adjustment for patients’ age, FIGO stage, and treatment, HPV16 was independently associated with better OS (HR = 0.36, 95% CI: 0.18, 0.74; *P* = 0.005) (Table 3). Infection with the five alpha-9 types was independently associated with better OS (HR = 0.17, 95% CI: 0.08–0.37; *P* < 0.001) and DFS (HR = 0.32, 95% CI: 0.17–0.59; *P* < 0.001). Further analysis suggested that HPV52/33/31/58 group had more favorable OS (HR = 0.12, 95% CI: 0.02–0.57; *P* = 0.008) and DFS (HR = 0.21, 95% CI: 0.06, 0.77; *P =* 0.018) (Additional file 1: Table S2). Given the limitation of statistical power, we did not find meaningful results when analyzing these four types individually.

To better understand the effect of HPV genotype on cervical cancer survival, stratified analyses based on age, FIGO stage, and treatment were performed. Although the protective effects of HPV16 and alpha-9 were more evident among those with FIGO stage III/IV and those receiving primary RT and/or CT, no significant difference was detected between subgroups (homogeneity test *P* > 0.05 for all) (Additional file 1: Table S3).

## Discussion

Despite recent progress in multimodal treatments, the clinical outcome of cervical cancer remains unfavorable. TNM or FIGO classification based on cervical pathology has insufficient predictive ability, because significant differences in survival are often observed for the same stage. Thus, it is highly necessary to explore additional biomarkers for the identification of a more effective therapeutic strategy against cervical cancer. In this study, we investigated prognostic value of HPV genotype for patients with cervical cancer. A total of 12 HR types were identified and HPV16 positivity was independently associated with lower risk of cervical cancer death than the group of the other 11 HR types. In addition, alpha-9 species including five HR types (16, 52, 33, 31, and 58) was a predictor of better survival compared with alpha-7 species group including the other five HR types (18, 39, 59, 68, and 45).

Substantial differences in risk for high-grade cervical intraepithelial neoplasia (CIN) and cervical cancer have been revealed between HR HPV types, in which HPV16 and HPV18 confer the highest risk [11, 12] However, the relationship between HPV genotype and cervical cancer prognosis has been controversial. Plich et al. identified HPV16 infection as a poor prognostic factor in 204 patients treated by primary radical hysterectomy and pelvic lymphadenectomy [4]. Conversely, another study showed that HPV16 positivity was significantly associated with improved prognosis in the whole series of cervical AC/ASC and also in subgroup receiving primary RT/CCRT [13]. In our study, the results supported the hypothesis that HPV16 has a favorable impact on the prognosis of cervical cancer. Further, we demonstrated that HPV16-related alpha-9 species significantly lowers the risk of cervical cancer-related death and recurrence/metastasis than the alpha-7 species, which was consistent with a previous study in patients undergoing primary radiotherapy [14]. Moreover, although several studies found that HPV18 positivity was associated with poorer prognosis of patients receiving primary surgery [5, 15, 16], other studies [17, 18] and ours failed to support the relationship. Given the much lower prevalence of HPV18 in cervical cancer than HPV16, independent studies with large sample size are needed to assess the impact of HPV18 on patients’ prognosis. In addition, because the HPV genotyping kit used in this study does not cover HPV67 and 70, which are high-risk types for cervical cancer, the impact of HPV67 and 70 on prognosis remains to be determined.

The underlying mechanisms that result in the tumors caused by HPV16 and the alpha-9 species being less aggressive are still undetermined. Interestingly, HPV status has been recognized as a strong and independent factor for favorable survival of patients with oropharyngeal cancer (OPC) [19, 20]. According to a systematic review, HPV prevalence was 35.6% (95% CI: 32.6–38.7%) in OPC specimens, and HPV16 accounted for a larger majority of HPV-positive OPC (86.7%; 95% CI, 82.6–90.1%) [21]. A better response to chemotherapy and radiation was observed for HPV-positive OPC [22–24]. In a worldwide survey of HPV genotype in cervix cancer, 61% of tumors were positive for HPV16 and 83% were positive for the alpha-9 species [3], similar to the data in our study. In vitro studies have revealed significant differences in biological behaviors between HPV types. For example, HPV16 is associated with a higher level of tumor apoptosis than HPV18, affording one possible explanation for more radiosensitive cervical cancer with HPV16 [25]. In addition, HR-HPV E6 proteins could interact with cellular PDZ domain-containing proteins to promote cell immortalization, invasion, and epithelial-to-mesenchymal transition (EMT) characteristics [26, 27]. There are significant differences in the interactions of HPV16 and HPV18 E6 with the PDZ domain-containing proteins, because a critical difference exists in the amino acid residue at the PDZ-binding motifs of the two E6 proteins [28]. This difference exists not only between HPV16 and HPV18, but also between the alpha-9 and alpha-7 species. Whether the variation in the PDZ domain-binding capacities determines the observed differential therapeutic response is worth additional exploration.

## Conclusions

Our results demonstrated the independent prognostic value of HPV genotype in cervical cancer. HPV genotyping could potentially help to stratify cervical cancer patients for more effective therapeutic regimens. Patients with alpha-9-caused cervical cancer may receive less aggressive therapy to reduce side effects, while those with alpha-7 positivity may require more aggressive treatment and closer monitoring. Identifying the mechanisms by which the alpha-7 species leads to a poorer prognosis could help to improve the outcome of cervical cancer patients.

## **Additional file**

Additional file 1: Table S1. Multiple HPV infections in cervical cancer patients. This table is the supporting information for line 169–171. **Table S2.** Survival analysis of the alpha-9 types in cervical cancer patients. This table is the supporting information for line 193–196. **Table S3.** Stratified analysis of HPV genotype and cervical cancer survival. This table is the supporting information for line 198–203. (DOCX 40 kb)

## Abbreviations

AC: Adenocarcinoma
ASC: Adeno-squamous carcinoma
CCRT: Concurrent chemoradiotherapy
CRT: Chemoradiotherapy
CT: Chemotherapy
DFS: Disease free survival
EMT: Epithelial-to-mesenchymal transition
HPV: Human papillomavirus
HR: Hazard ratio
OPC: Oropharyngeal cancer
OS: Overall survival
RT: Radiotherapy
