# Supplementary material for: Independent prognostic role of human papillomavirus genotype in cervical cancer
Source: BMC Infect Dis. 2017 Jun 5;17:391. doi: 10.1186/s12879-017-2465-y (PMC5460478; doi:10.1186/s12879-017-2465-y)
Supplement: Additional file 1: Table S1. — Multiple HPV infections in cervical cancer patients. This table is the supporting information for line 169–171. Table S2. Survival analysis of the alpha-9 types in cervical cancer patients. This table is the supporting information for line 193–196. Table S3. Stratified analysis of HPV genotype and cervical cancer survival. This table is the supporting information for line 198–203. (DOCX 40 kb) [file 12879_2017_2465_MOESM1_ESM.docx]

**Supporting information**

| Table S1. Multiple HPV infections in cervical cancer patients | | |
| --- | --- | --- |
| HPV types | Frequency | Percent (%) |
| 16+52 | 6 | 15.79 |
| 16+33 | 4 | 10.53 |
| 16+33+58 | 3 | 7.89 |
| 16+58 | 3 | 7.89 |
| 16+18 | 2 | 5.26 |
| 18+52 | 2 | 5.26 |
| 16+18+33 | 1 | 2.63 |
| 16+18+66 | 1 | 2.63 |
| 16+33+52+58 | 1 | 2.63 |
| 16+33+53 | 1 | 2.63 |
| 16+39 | 1 | 2.63 |
| 16+39+52 | 1 | 2.63 |
| 16+39+58+66 | 1 | 2.63 |
| 16+45 | 1 | 2.63 |
| 16+52+56 | 1 | 2.63 |
| 16+52+58 | 1 | 2.63 |
| 16+52+58+59 | 1 | 2.63 |
| 16+52+66 | 1 | 2.63 |
| 16+68 | 1 | 2.63 |
| 18+33 | 1 | 2.63 |
| 33+58 | 1 | 2.63 |
| 33+59 | 1 | 2.63 |
| 39+52 | 1 | 2.63 |
| 52+68 | 1 | 2.63 |
| Total | 38 | 100.00 |

| Table S2. Survival analysis of the alpha-9 types in cervical cancer patients | | | | | | |
| --- | --- | --- | --- | --- | --- | --- |
| HPV types | | Overall Survival | |  | Disease Free Survival | |
|  |  | Adjusted HR | *P* ^a^ |  | Adjusted HR | *P* ^a^ |
|  |  | (95% CI) ^a^ |  |  | (95% CI) ^a^ |  |
| HPV16/52 | |  |  |  |  |  |
|  | No | 1.00 |  |  | 1.00 | 0.107 |
|  | Yes | 0.25 (0.12, 0.52) | < 0.001 |  | 0.59 (0.31, 1.12) |  |
| HPV16/33 | |  |  |  |  |  |
|  | No | 1.00 |  |  | 1.00 | 0.306 |
|  | Yes | 0.33 (0.16, 0.68) | 0.003 |  | 0.72 (0.38, 1.35) |  |
| HPV16/31 | |  |  |  |  |  |
|  | No | 1.00 |  |  | 1.00 | 0.514 |
|  | Yes | 0.38 (0.19, 0.78) | 0.010 |  | 0.81 (0.44, 1.51) |  |
| HPV16/58 | |  |  |  |  |  |
|  | No | 1.00 |  |  | 1.00 | 0.402 |
|  | Yes | 0.35 (0.17, 0.71) | 0.004 |  | 0.77 (0.41, 1.43) |  |
| HPV52/33/31/58 ^†^ | |  |  |  |  |  |
|  | No | 1.00 | 0.008 |  | 1.00 | 0.018 |
|  | Yes | 0.12 (0.02, 0.57) |  |  | 0.21 (0.06, 0.77) |  |
| ^a^ Adjusted for age, FIGO stage, treatment in the Cox regression models. | | | | | | |
| ^†^ HPV16-positive cases were excluded in the analysis. | | | | | | |

| Table S3. Stratified analysis of HPV genotype and cervical cancer survival | | | | | | | | | | | |  |
| --- | --- | --- | --- | --- | --- | --- | --- | --- | --- | --- | --- | --- |
| Variable | | HPV16 and overall survival | | |  | Alpha-9 and overall survival | | |  | Alpha-9 and disease free survival | | |
|  |  | Adjusted HR (95% CI) ^a^ | *P* ^a^ | *P* ^b^ |  | Adjusted HR (95% CI) ^a^ | *P* ^a^ | *P* ^b^ |  | Adjusted HR (95% CI) ^a^ | *P* ^a^ | *P* ^b^ |
|  |  |  |  |  |  |  |  |  |  |  |  |  |
| Age, years | |  |  |  |  |  |  |  |  |  |  |  |
|  | ≤50 | 0.50 (0.19, 1.31) | 0.155 | 0.517 |  | 0.13 (0.04, 0.40) | <0.001 | 0.791 |  | 0.36 (0.14, 0.90) | 0.029 | 0.605 |
|  | >50 | 0.28 (0.09, 0.81) | 0.019 |  |  | 0.17 (0.06, 0.53) | 0.002 |  |  | 0.24 (0.10, 0.60) | 0.002 |  |
| FIGO stage ^†^ | |  |  |  |  |  |  |  |  |  |  |  |
|  | I/II | 0.47 (0.18, 1.26) | 0.134 | 0.476 |  | 0.21 (0.08, 0.56) | 0.002 | 0.433 |  | 0.41 (0.19, 0.89) | 0.024 | 0.372 |
|  | III/IV | 0.24 (0.08, 0.74) | 0.013 |  |  | 0.09 (0.02, 0.38) | 0.001 |  |  | 0.21 (0.07, 0.60) | 0.004 |  |
| Treatment ^‡^ | |  |  |  |  |  |  |  |  |  |  |  |
|  | Primary surgery | 0.47 (0.03, 7.51) | 0.591 | 0.967 |  | 0.15 (0.01, 2.68) | 0.199 | 0.977 |  | 0.69 (0.14, 3.42) | 0.648 | 0.61 |
|  | Primary RT/CT | 0.39 (0.19, 0.79) | 0.010 |  |  | 0.17 (0.08, 0.39) | <0.001 |  |  | 0.26 (0.13, 0.52) | <0.001 |  |
| ^†^ FIGO, International Federation of Gynecology and Obstetrics.  ^‡^ RT, radiotherapy; CT, chemotherapy.  ^a^ Adjusted for age, FIGO stage except for the stratification factor in Cox regression model. | | | | | | | | | | | | |
| ^b^ *P* value for the homogeneity test. | | | | | | | | | | | | |
